# First study of the detection of Human Herpes Virus-8 and major blood-borne viruses in iranian patients with SLE: A cross-sectional study

**DOI:** 10.1016/j.nmni.2024.101445

**Published:** 2024-06-20

**Authors:** Leila Soltani, Ava Hashempour, Javad Moayedi, Maryam Feili, Zahra Musavi, Mohammad Ali Nazarinia

**Affiliations:** a HIV/AIDS Research Center, Institute of Health, Shiraz University of Medical Sciences, Shiraz, Iran; b Geriatric Research Center, Shiraz University of Medical Sciences, Shiraz, Iran

**Keywords:** Autoimmune disease, Viral infections, SLE, Human herpesvirus-8

## Abstract

**Background:**

Systemic lupus erythematosus (SLE) is an autoimmune disease caused by genetic and environmental factors such as viral infections. Genomic and serologic tests were applied to detect significant blood-borne viruses in SLE patients to determine whether there was a possible association between viral infections and SLE.

**Methods:**

Antibodies (Abs) against HHV-8, HCMV, EBV, HIV, HBV, and HCV in SLE patients suffering from SLE were assessed by ELISA. In addition, HHV-8 DNA and HIV-1 RNA were quantified by real-time PCR, and the HCV and HBV genomes were detected using nested PCR.

**Results:**

Compared to those in the control group, a high prevalence of anti-HHV-8 (p < 0.0001), anti-HCMV (p = 0.014), and anti-EBV (p = 0.017) Abs was detected in SLE patients. HHV-8, HIV, HCV, and HBV genomic tests were negative in both groups, while only 1.1 %, 2.2 %, and 1.1 % of SLE patients were positive for anti-HIV, anti-HCV Abs, and HBsAg, respectively. The most frequent major complaint in patients was arthralgia (76.7 %).

**Conclusions:**

The increased prevalence of anti-HHV-8 Abs may not be related to the natural history of infection but to molecular mimicry. Increased anti-HCMV and anti-EBV Abs may also be associated with the development of SLE and may play direct or indirect roles in such infections or molecular mimicry. Since arthralgia is the most common symptom in SLE patients, the presence of these symptoms in any patient is a suggestive clue for the diagnosis of SLE. Defining the typical pattern of SLE in divergent nations with distinct environmental and geographical factors can be beneficial for obtaining a prompt diagnosis.

## 1 Introduction

Systemic lupus erythematosus (SLE) is a chronic systemic autoimmune disease with varied clinical manifestations. Clinically, heterogeneous manifestations, including hair loss, skin lesions, hematocytopenia, neuropsychiatric involvement and other internal organ damage, have been reported in various patient populations. According to a large population-based study by the Iranian Rheumatic Diseases Control Society, the prevalence of lupus in Iran is estimated to be 40 per 100,000 people [1]. The complex pathogenesis of SLE has not been completely elucidated; however, the mechanisms responsible for this phenomenon can be summarized as follows: imbalance of both humoral and cellular immunity, which comprises hyperactivity of B cells producing autoantibodies, excess cytokines, and defective T regulatory cell function that fails to maintain self-tolerance. These abnormalities eventually culminate in immune-mediated tissue damage and inflammation [2].

The etiology of SLE is still not fully understood; however, there is conspicuous evidence illustrating that both environmental and genetic factors contribute to SLE development. It seems that genetic factors take part in the stimulation of environmental factors; however, a direct “cause and effect” relationship has yet to be demonstrated. For decades, research groups have attempted to identify the environmental agents that contribute to the development of SLE, including viral infections [2,3]. Viral infections are a significant threat to public health and are a great threat to human society. Several reports have revealed the importance of viral infections, either as main targets or cofactors, in both the development and progression of the pathological characteristics of SLE. Several reports illustrate that infections are regarded as one of the leading causes of hospitalization among SLE patients, significantly contributing to morbidity and mortality in this population [4].

Although immune responses are crucial for suppressing viral infections, molecular mimicry between host proteins and viral antigens, a non-modulated immune response and an exaggerated hypersensitivity reaction may result in the production of numerous cross-reactive autoantibodies that lead to tissue damage in patients with SLE. Because of the large pool of autoantigens and the immunological and clinical heterogeneity of SLE patients, diverse viral proteins may participate in molecular mimicry [3]. Generally, two results can be reached due to the production of cellular homologues, which are mistaken for viral products. The first is structural mimicry; for example, Epstein‒Barr virus (EBV) nuclear antigen-1 (EBNA-1) mimics human autoantigens and contributes to the onset of autoimmunity [5]. The second is functional mimicry; for example, HHV-8 expresses homologues of human proteins to protect cells from apoptosis [6]. In SLE pathogenesis, striking evidence implies that human cytomegalovirus (HCMV) and other agents can be prime candidates for treating vascular damage. HCMV has various interactions with the host immune system, which may be conducive to conferring SLE-specific clinical features [7]. Other studies have implicated hepatitis B virus (HBV) and hepatitis C virus (HCV), as well as human immunodeficiency virus (HIV), as possible environmental factors involved in the onset and/or exacerbation of SLE.

The current study is the first to investigate the relationship between anti-HHV-8 Abs and SLE among Iranian patients. Additionally, this is the first simultaneous investigation of the seroprevalence of major blood-borne viruses and the genomic detection of HHV-8, HIV, HCV, and HBV in SLE patients. Considering the unclear role of viral infections in SLE patients, this investigation was conducted to identify a possible link between SLE and blood-borne viruses by the following assessments in Iranian patients with SLE: 1: the first evaluation of the seroprevalence of HHV-8; 2: genomic detection of HHV-8, HCV, HIV, and HBV infections; 3: evaluation of the seroprevalence of HHV-8, EBV, HCMV, HIV, HBV, and HCV infections; and 4: evaluation of SLE manifestations and its possible relationship with other factors. This study may provide new insight into the relationship between viral infections, especially HHV-8 infection, and SLE.

## 2 Materials and methods

### 2.1 Patients

In this cross-sectional study from 2021 to 2022, 90 adult Iranian patients met the American College of Rheumatology (ACR) requirements for the diagnosis of SLE. All participants with an active record and confirmed history of SLE routinely visited the Rheumatology Clinic of Hafez Hospital, affiliated with Shiraz University of Medical Sciences, Shiraz, Iran. In studies related to SLE, control groups play a crucial role in comparing various factors and outcomes. Age and sex significantly influence the clinical presentation, progression, and outcomes of systemic lupus erythematosus patients. Females are disproportionately affected by SLE [8,9]; however, males often experience more severe disease manifestations [10]. Pediatric-onset SLE is more severe than adult-onset SLE [11,12]. Ethnic disparities further complicate the management landscape [13]. Although advancements in treatment have improved survival rates overall, targeted approaches considering age-, sex-, and ethnicity-specific factors are essential for optimizing patient outcomes. In this study, the control group included 90 adult Iranian participants of both genders who did not have a family history of SLE or who tested positive for ANA IgG antibodies. In addition, they were matched with the patients to eliminate the influences of confounding factors, including sex and age. Moreover, all individuals had the same ethnic genetics as they were originally from Iran, and they belonged to the Middle Eastern ethnicity to exclude ethnic-specific genetics. The participants also lived in the same city to exclude environmental confounders, including weather, etc., as much as possible. The exclusion criteria were a history of blood transfusion or transfusion of any blood products and receiving the HBV vaccine in both patients and controls. Patient data were collected from medical records, and based on the Declaration of Helsinki and its later amendments, written informed consent was obtained from all participants.

### 2.2 Specimen collection

Five-millilitre peripheral blood samples were collected in nonheparinized tubes and centrifuged at 3000 RPM for 5 min. Finally, the serum fraction was separated, labelled, and stored at −80 °C.

### 2.3 Genomic DNA extraction, HHV-8 real-time PCR assay, and HBV PCR

Using the Invisorb spin virus DNA mini kit (Stratec Biomedical, Germany), high-quality viral DNA was extracted according to the manufacturer's protocol. The HHV-8 viral load was assessed by a quantitative real-time assay (Genesig standard kit, primer design, UK). This kit includes primers that exhibit 100 % homology with more than 95 % of the reference sequences for HHV-8, such as U75698, U93872, and AF148805. The reaction mixture consisted of 10 μL of 2X qPCR master mix, 1 μL of HHV8 primer/probe mix, and 5 μL of DNA template, and the total volume was adjusted to 20 μL using RNase/DNase-free water. Each set of real-time PCRs contained appropriate negative and positive controls, and standard precautions were adopted to avoid contamination. The real-time PCR assay was performed on a 7500 PRISM machine (Applied Biosystems) under the following conditions: 95 °C for 2 min, followed by 50 cycles at 95 °C for 10 s and 60 °C for 60 s. The amplification of the target sequence was detected through the FAM channel.

HBV Nested-PCR was performed using specific primers for the surface region (Table 1), as described previously [14].

**Table 1:** List of HCV and HBV primers used in this study and the thermal cycling conditions used for nested PCR.

Primers		Sequence	Location	Products length (bp)	PCR (I) and (II) Programs
**HCV**	**Outer Forward**	AGCGTCTAGCCATGGCGT	74–91	264	94 (2 min) 35 cycles 94 (30 s) 40 (30 s) 72 (55 s) 72 (5 min)
	**Outer Reverse**	GCACGGTCTACGAGACCT	321–338		
	**Inner Forward**	GTGGTCTGCGGAACCGG	143–159	174	
	**Inner Reverse**	GGGCACTCGCAAGCACCC	298–317		
**HBV**	**Outer Forward**	GAGTCTAGACTCGTGGTGGACTTC	246–269	447	94 (5 min) 35 cycles 94 (1 min) 60 (1 min) 72 (2 min) 72 (10 min)
	**Outer Reverse**	AAATKGCACTAGTAAACTGAGCCA	670–693		
	**Inner Forward**	CGTGGTGGACTTCTCTCAATTTTC	257–280	419	94 (5 min) 30 cycles 94 (1 min) 44 (1 min) 72 (1.5 min) 72 (10 min)
	**Inner Reverse**	GCCARGAGAAACGGRCTGAGGCCC	650–673		

### 2.4 RNA extraction, HIV viral load, and HCV RT‒PCR

RNA was extracted from serum by the QIAamp Viral RNA Mini Extraction Kit (Qiagen, Germany), which was followed by an assessment of HIV viral load using the AltoStar® HIV RT‒PCR test kit (Hamburg, Germany). For the detection of HCV RNA in serum, cDNA synthesis and nested PCR were performed using a specific primer set for HCV-ULR [15] (Table 1). GAPDH was used as the positive control for both the RT‒PCR and PCR assays.

### 2.5 Immunological evaluations

ELISA was performed to evaluate the seropositivity of specific Abs against HCMV (HCMV-IgG, Dia. Pro., Italy), EBV (EBV-VCA-IgG, Dia. Pro, Italy), HHV-8 (HHV-8 IgG, Advanced Biotechnologies Inc., USA), and HBV (HBsAg, Dia. Pro., Italy), HCV (HCVAb, Dia. Pro., Italy), and HIV (HIVAb, Dia. Pro, Italy). It should be noted that the HHV-8 IgG ELISA kit was designed to detect specific IgG antibodies to lytic antigens of HHV-8 in human serum or plasma. Stop solution was added to terminate the enzymatic reaction, and the absorbance of the samples was measured using an ELISA-Reader (Epoch Microplate Spectrophotometer, Biotek, USA). The ELISA cut-off value was calculated according to the manufacturer's recommendations.

The levels of autoantibodies, including anti-nuclear antibody (ANA), anti-double stranded DNA (anti-dsDNA), and anti-cardiolipin antibody (ACA), were determined by ELISA with a commercial kit (LifeSpan BioSciences, USA) according to the manufacturer's instructions.

### 2.6 Statistical analysis

The median (Q1 and 3), frequency (%), Spearman coefficient correlation, and chi-squared test were used for quantitative data. All reported probabilities (*P values*) less than 0.05 were considered to indicate statistical significance. Data management was performed using SPSS version 20.0.

## 3 Results

**Fig. 1:**
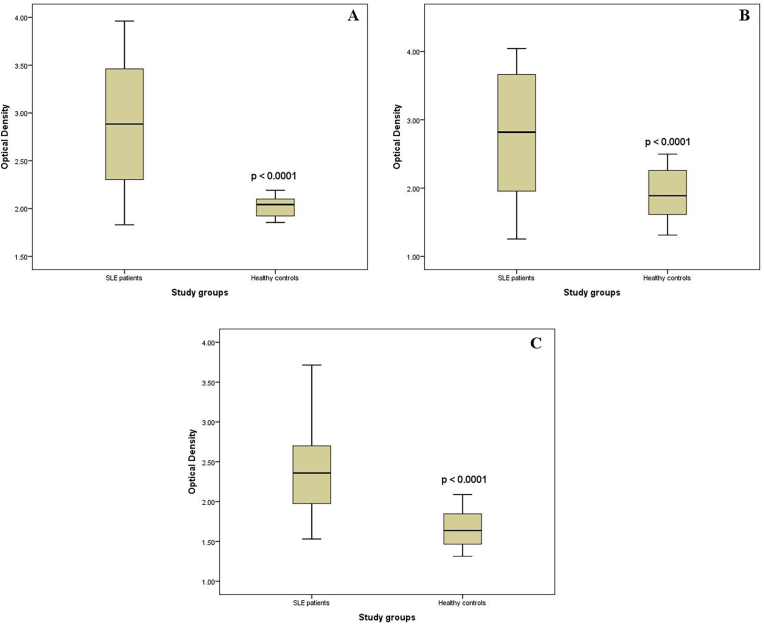
Optical density of antibodies against HHV-8 (A), HCMV (B), and EBV (C) measured by ELISA. SLE: systemic lupus erythematosus; HHV-8: human herpesvirus-8; HCMV: human cytomegalovirus; EBV: Epstein–Barr virus; ELISA: enzyme-linked immunosorbent assay.

As shown in Table 2, the patients with SLE consisted of 80 (88.9 %) females and 10 (11.1 %) males, while the control group was composed of 79 (87.8 %) females and 11 (12.2 %) males; thus, the two groups were sex-matched (p > 0.05). Furthermore, the median ages of healthy individuals and patients with SLE individuals were 37.9 (18–54) and 38 (18–50) years, respectively, indicating age-matched groups (p = 0.245). Approximately 33 % of SLE patients had a family history of autoimmune diseases. A significant difference in the HHV-8 IgG Ab frequency between patients and controls (51.1 % vs. 5.6 %) was found (p < 0.05). Interestingly, HHV-8 genomic DNA was not detected in the SLE patients or healthy controls, whereas a high-sensitivity real-time PCR assay with two copies in ml was performed in this study. The difference in the frequency of positive Abs for both HCMV and EBV viruses between patients and controls was significant (p = 0.014 and p = 0.017, respectively). Compared to those in the healthy population, the optical density (OD) of anti-HHV-8, anti-HCMV, and anti-EBV Abs was significantly (p < 0.0001) greater in SLE patients (Fig. 1).

**Table 2:** Demographic, serologic, and genomic data of the studied individuals.

Variables		Control (n = 90)	Patients (n = 90)	p value
**Sex**	Male, n (%)	11 (12.2 %)	10 (11.1 %)	p > 0.05
	Female, n (%)	79 (87.8 %)	80 (88.9 %)	
**Age**	Median (^≠^Min-^α^Max)	37.9 (18–54)	38 (18–50)	p = 0.245
^**€**^**HHV-8 IgG**	Positive, n (%)	5 (5.6 %)	46 (51.1 %)	p < 0.0001
	Negative, n (%)	85 (94.4 %)	44 (48.9 %)	
^**∞**^**CMV IgG**	Positive, n (%)	83 (92.2 %)	90 (100 %)	p = 0.014
	Negative, n (%)	7 (7.8 %)	Undetectable	
^**£**^**EBV IgG**	Positive, n (%)	68 (75.6 %)	81 (90 %)	p = 0.017
	Negative, n (%)	22 (24.4 %)	9 (10 %)	
**HHV-8 Viral Load**		Undetectable	Undetectable	–
^**±**^**HBV**^**ү**^**DNA**		Undetectable	Undetectable	–
^**β**^**HCV**^**×**^**RNA**		Undetectable	Undetectable	–
^**π**^**HBsAg**		Undetectable	1 (1.1 %)	p > 0.05
= **HCVAb**		Undetectable	2 (2.2 %)	p > 0.05
^**¥**^**HIVAb**		Undetectable	1 (1.1 %)	p > 0.05

^≠^Minimum, ^α^Maximum, ^€^Human Herpesvirus-8 Immunoglobulin G, ∞Cytomegalovirus Immunoglobulin G, £Epstein Barr Virus Immunoglobulin G, *Not found, ±Hepatitis B virus, ^ү^Deoxyribonucleic acid, ^β^Hepatitis C virus, ^×^Ribonucleic acid, ^π^Hepatitis B surface antigen, = Antibody, ^¥^Human immunodeficiency virus.

### 3.1 Immunological and clinical data

**Fig. 2:**
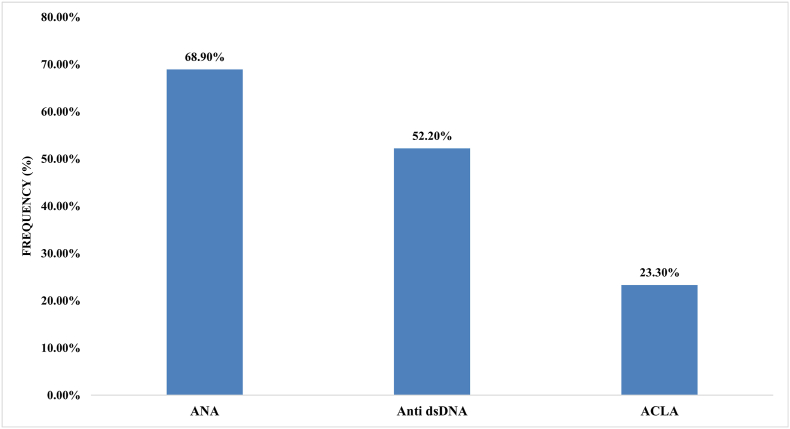
Frequency of autoantibodies in SLE patients. SLE: systemic lupus erythematosus; ANA: antinuclear antibodies; dsDNA: double-stranded DNA.

**Fig. 3:**
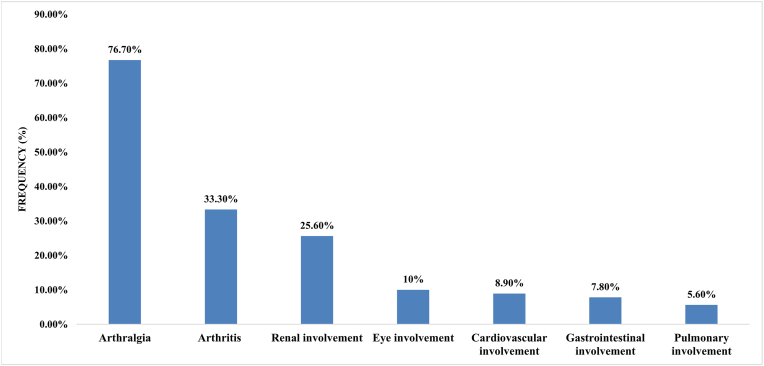
Frequency of manifestations in SLE patients. SLE: systemic lupus erythematosus.

ANA-positive antibodies were the most common autoantibody (68.9 %) among the SLE patients, followed by anti-dsDNA Abs (52.2 %) and ACAs (23.3 %) (Fig. 2). As shown in Fig. 3, arthralgia was the most common manifestation (76.7 %), followed by arthritis (33.3 %). In addition, the frequency of each involvement in women and men is shown in Table 3. Arthralgia was the most frequent representation in 77.5 % of women and 70 % of men. Only anti-HHV-8 Abs were significantly associated with arthralgia and positive anti-dsDNA Abs and ANAs (Table 4). In contrast, there was no association between other viral infections and organ involvement or positive results for autoimmune disorders (p > 0.05). We can also postulate that there is some cross-reactivity between autoantibodies and HHV8. Only one patient in the case group was positive for both HHV-8 and autoantibodies, while none of the individuals in the control group were positive for autoantibodies. A larger sample size could provide a more comprehensive understanding of the potential cross-reaction between autoantibodies and HHV-8.

**Table 3:** Frequency of clinical symptoms in patients with SLE stratified by sex.

Clinical Signs	Female (n = 80)	Male (n = 10)	P Value
Cardiac involvement	8 (10 %)	NF^a^	p = 0.590
Gasterointestinal involvement	5 (6.2 %)	2 (20 %)	p = 0.173
Pulmonary involvement	4 (5 %)	1 (10 %)	p = 0.453
Arthrithis	28 (35 %)	2 (20 %)	p = 0.486
Argthralgia	62 (77.5 %)	7 (70 %)	p = 0.693
Renal involvement	20 (25 %)	3 (30 %)	p = 0.712
Eye involvement	7 (8.8 %)	3 (30 %)	p = 0.079

a NF: not found.

**Table 4:** The correlation of the clinical and laboratory characteristics of SLE patients who were seropositive for viral infections. Arthralgia was the most common clinical symptom in our SLE patients. All individuals were negative for HBsAg, HCVAb, and HIVAb.

Manifestation	Total, n (%)	^∞^HCMV IgG	^£^EBV-VCA IgG	^μ^HHV-8 IgG
**Arthralgia**	69 (76.7 %)	69 (100 %)	61 (88.4 %)	a 40 (58 %)
**Arthritis**	30 (33.3 %)	30 (100 %)	26 (86.7 %)	19 (63.3 %)
**Renal involvement**	23 (25.6 %)	23 (100 %)	22 (95.7 %)	13 (56.5 %)
**Ocular involvement**	9 (10 %)	10 (100 %)	9 (90 %)	5 (50 %)
**Cardiovascular involvement**	8 (8.9 %)	4 (100 %)	4 (100 %)	4 (50 %)
**Gastrointestinal tract involvement**	7 (7.8 %)	7 (100 %)	7 (100 %)	6 (85.7 %)
**Pulmonary involvement**	5 (5.6 %)	5 (100 %)	5 (100 %)	3 (60 %)

^∞^HCMV IgG: Human Cytomegalovirus Immunoglobulin G.

^£^EBV-VCA IgG: Epstein‒Barr Virus Viral Capsid Antigen Immunoglobulin G.

^μ^HHV-8 IgG: Human Herpesvirus-8 Immunoglobulin G.

a p < 0.05.

## 4 Discussion

In the first report from Iran, a significantly greater frequency (51.1 % vs 5.6 %) and higher OD levels of anti-HHV-8 Ab were found in patients with SLE than in healthy individuals. Although the OD and titre of antibodies are different, the data obtained indicate the appropriate antibody concentration [16]. In a study by Tung, a higher titre of anti-HHV-8 Ab was detected in patient samples. Here, all subjects were negative for the HHV-8 DNA genome, while some reports have shown a positive viral load in such patients [3]. There are two probable explanations for the high HHV-8 seropositivity (51.1 %) in Iranian SLE patients with a negative HHV-8 viral load. The first is the production of anti-HHV-8 Ab in either the lytic or latent phase of HHV-8 infection, which results in an increased seroprevalence of HHV-8 in SLE patients. This explanation is in agreement with the meta-analysis supporting the hypothesis that HHV-8 and EBV infections predispose patients to the development of SLE. Furthermore, one of the factors that predisposes SLE patients to HHV-8 infection is the routine use of immunosuppressive agents in SLE treatment procedures [17], which increases susceptibility to reactivation of lytic HHV-8 or infection with HHV-8 and other blood-borne viruses, such as viral hepatitis and HIV. However, this hypothesis may not be plausible in this study since the prevalence of anti-HHV8 Abs in the general Iranian population is quite low and has been reported to reach 3.6 % [6]. Moreover, the lack of blood transfusion history or high-risk issues in the studied SLE patients indicated that the high prevalence of anti-HHV-8 Abs may not be related to true positive HHV-8 infection. Furthermore, all the SLE patients were in the active phase of the disease, while the HHV-8 genome was negative in all the patients according to the very sensitive kit, which detects even two copies of DNA per ml. As a result, the present findings shifted our conclusion to mimicry with host proteins. Several reports have indicated that viral infections might trigger autoimmune diseases through bystander activation, epitope spreading, adjuvant effects, and molecular mimicry, as described in the following sentences [5].

The other viral infections studied in this report were HCMV and EBV. Here, significantly greater frequencies and OD levels of both anti-HCMV and anti-EBV Abs were detected in patients with SLE than in the control group. The findings of this study concur with some reports regarding the greater frequency and titre of HCMV-IgG Ab in patients with SLE than in controls [18]. Additionally, the higher prevalence of EBV infection in patients with SLE than in controls (90 % versus 75.6 %) was in line with the findings of some research groups [19].

Elevated Ab titres against EBV antigens and cytomegalovirus were reported to be significantly greater in patients with SLE than in healthy controls [20]. HCMV and EBV are ubiquitous viruses in the general population in Iran; therefore, such infections are likely to occur in patients with SLE. Thus, it is difficult to represent an apparent assumption of the causal link between HCMV and EBV infections and SLE. However, we suspected that the higher prevalence and OD levels of anti-HCMV and EBV Abs in SLE patients indicate indirect markers of increased HCMV and EBV reactivation and molecular mimicry or an increased incidence of infection long before the onset of SLE rather than secondary infections resulting from an impaired immune system or the use of immunosuppressive drugs [3].

The other viral infections investigated in this study were HBV and HCV. Compared with the healthy individuals who were negative for HCV Ab and HBsAg, the SLE group appeared to have a greater frequency of anti-HCV Ab (2.2 %) and HBsAg (1.1 %), but these differences were not significant (P > 0.05). Since both groups had negative HBV and HCV molecular tests, a causal link might not exist between HCV/HBV and Iranian SLE patients. Similar to another report [21], the rate of HBsAg positivity in SLE patients was lower than that in the control group, but the difference was not significant, suggesting a protective role of SLE autoimmunity through a proinflammatory environment that protects against HBV infection in SLE patients [21].

The last infection that was assessed in the current study was HIV, and there are no reports on HIV viral load and anti-HIV Abs in Iranian SLE patients. The present data revealed a negative HIV viral load in both the control and patient groups. Likewise, the control group was negative for anti-HIV Ab, while only 1 % of the SLE patients were anti-HIV Ab positive. Although HIV-infected patients can develop some autoimmune disorders with severe manifestations, there is a discrepancy in whether HIV infection can increase the incidence of SLE. Several theories have been proposed to explain the lower prevalence of HIV in SLE patients [22]. The interleukin (IL)-16 level is greater in SLE patients than in healthy controls, which suggests that IL-16 plays a protective role against HIV because it inhibits HIV replication in vitro.

Another part of this study investigated clinical and laboratory factors. In this report, the most frequent clinical manifestation in SLE patients was arthralgia (76.7 %), which was in accordance with previous reports showing that arthralgia was the most common presentation in patients with SLE [23] but was a less common disorder than in other reports [24].

Renal involvement was present in 25.6 % of patients, at least two times less than in another region of Iran that is mountainous with inhabitants of different races [24]. Climatic factors or genetics may affect the incidence of various presentations of lupus. Renal manifestations are more frequent in Indians, Africans, and Chinese people and less prevalent in Arabs, Caucasians, Ricans, and Puerto people. In addition to the intensity of sunlight and temperature, nutritional and socioeconomic factors significantly influence the frequency of renal involvement [25]. The prevalence of ocular involvement was 10 % (Table 1), which was similar to that reported by Barcia-Sixto et al. [26]. CVD is among the leading causes of morbidity and mortality in SLE patients and is present in at least 50 % of SLE patients. Here, CVD was present in 8.9 % of the SLE patients, and these data are similar to those of other studies from Iran [25]; however, more SLE patients suffer from CVD in other studies from Iran or other countries [23,26].

Despite the high prevalence of liver abnormalities in SLE patients, gastrointestinal manifestations presented at a low level (7.8 %) in our patients, which was similar to the findings of another study from the same region of Iran; however, data from other countries revealed that 20–50 % of SLE patients had gastrointestinal involvement [27].

Among the various presentations, the pulmonary feature rate was the lowest, presenting at 5.6 % in SLE patients. This prevalence was similar to that in another report [28]; however, pulmonary manifestations were greater in other studies in Iran [24,25].

Our findings revealed that 30 % of the SLE patients were negative for ANA, which was in agreement with the findings of studies from Iran [24], while the percentage of patients who were positive for ANA in other regions of Iran was 70–98 % [29,30]. Similar to other studies, ANA positivity was more frequent in families with a positive history of autoimmune disorders, which may highlight the importance of ANA antibodies in the primary detection of autoimmune disease [31]. Another immunological marker analysed in our patients was anti-dsDNA (52.2 %), which was lower than the results of some previous studies [25,28,32]. According to our data, 23.3 % of the SLE patients were ACA positive, which was similar to that of Europeans [33] but higher than that of Chinese individuals [34] and lower than that of Middle Eastern countries [35]. In our opinion, there was a significant relationship between positive anti-HHV-8 Ab and anti-dsDNA (0.012) and ANA (0.004). These data suggest that a causal link might exist between HHV-8 viremia and the development of SLE. As our previous study showed a high prevalence of HHV-8 Ab (67.8 %) in systemic sclerosis (SSc) [6], it can be proposed that the increased anti-HHV-8 Ab in both SLE and SSc patients could be related to autoimmune disease; however, further studies are needed to evaluate this hypothesis.

Here, 33 % of the SLE patients had a positive family history of autoimmune disease, which agreed with reports of familial aggregation of SLE. Nonetheless, positivity for family history was not associated with any signs in our SLE patients, which was in contrast with other reports [32]. Finally, the female-to-male ratio (9/1) in SLE patients was approximately equal to that in other reports [19] that showed a profound sex bias in SLE patients, which may be due to the sex hormones that affect SLE development [36]. Among the various manifestations in our SLE patients, arthralgia was the most common clinical symptom in women (77.5 %) and men (70 %), followed by arthritis (35 %) and renal/eye involvement (30 %) in women and men, respectively.

Discrepancies in the frequency of bloodborne viruses, presence of autoantibodies, and clinical features among various studies may be due to differences in sample size; age, type and quality of the kit; immunosuppressed status; treatment status; severity and duration of the disease; study design; technique and sensitivity of the kits; nonuniform SLE definitions; and inherent differences in population structure, such as sex distribution; socioeconomic status; environmental, immunologic and cytokine levels; hormonal and genetic risk factors; probability of having a family history of SLE; racial issues; geographical region; and environmental exposures.

The generalizability or external validity of research findings on lupus infection is a critical concern given the heterogeneity inherent in SLE. Studies have highlighted the increased risk of infections, particularly bacterial infections, in patients with SLE [37,38]. Factors such as age, neutrophil count, disease activity score, and medication history have been identified as predictors of severe infections in SLE patients [39,40]. Additionally, the development of clinical prediction models using readily available clinical and laboratory data has shown promise in assessing the risk of hospital-acquired bacterial infections in SLE patients [41]. In addition, a review on the causes of mortality in patients with SLE identified infections as a significant contributor to cardiovascular complications and disease activity. This multifactorial nature necessitates comprehensive management strategies tailored to individual risk profiles, challenging the universal applicability of any single intervention [8]. These findings suggest that the results regarding lupus infection risk and outcomes can be generalized to similar patient populations, emphasizing the importance of early identification and management strategies to improve patient care and outcomes.

Here, the authors proposed some suggestions, as patients with SLE have a lower survival rate than the general population; therefore, those with positive Abs for blood-borne viruses should be considered in a particular category before initiating any treatment with immunosuppressants. In the complex immune setting of SLE, performing an HHV-8 serology assay at the time of diagnosis may be necessary to clarify the basic history of the infection. Furthermore, the increased seroprevalence of EBV, HCMV, and HHV-8 infections in SLE patients suggests active monitoring of such viruses via molecular tests in SLE patients to improve patient treatment outcomes. Moreover, bioinformatic studies are necessary to design new drugs against homologous antigenic epitope regions to prevent cross-reactivity and autoimmune disease. It seems that in various parts of Iran and different countries, the typical pattern of SLE may be unique; thus, designing a specific database for SLE can provide deeper insight into the diagnosis and treatment of this disease [42].

Despite some limitations in the present study, such as the lack of prior sample size estimation and lack of demographic and laboratory data, there has not yet been any study in Iran that simultaneously investigated the seroprevalence and molecular detection of HHV-8, EBV, HCMV, HIV, HBV, and HCV infections in Iranian SLE patients. Therefore, we included as many patients per group as possible. However, the general limitations of studies focusing on lupus patients include the retrospective nature of the research, incomplete or delayed vaccination statuses, lack of active screening of tuberculosis, differences in immunosuppressive therapy, socioeconomic factors, and ethnicity composition. Further studies with longer follow-up periods, larger sample sizes, and rare-frequency assessments of the seroprevalence of blood-borne viruses are warranted for a better understanding of the interactions between viral infection and autoimmunity in patients with SLE. In this study, serum was used for serological tests and (RT)-PCR; however, plasma or whole blood is usually more accurate to do PCR assay for HIV, HBV, HCV, and HHV-8. Therefore, underestimation of viral load and false negatives are possible with (RT)-PCR and False negative results are particularly likely for HHV-8 due to its latent phase in lymphocytes. Investigating the possible role of HHV-8 in other autoimmune diseases and comparing SLE manifestations in various regions is highly recommended.

## 5 Conclusions

According to the first report of ani-HHV-8 Ab in Iranian SLE patients, the high prevalence of anti-HHV-8 Ab in SLE patients with a negative DNA load may correspond to the production of cross-reactive Ab and the development of molecular mimicry because HHV-8 is not only common in the general Iranian population but also because none of the studied SLE patients had a history of blood transfusion. Consequently, this observation raises the possibility of an association between HHV-8 viremia and SLE development, which can facilitate the quick diagnosis of systemic lupus erythematosus. HHV-8 false-positivity was detected by ELISA in SLE patients, making it necessary to perform other confirmatory tests, such as molecular tests. Here, it was found that arthralgia was the most common clinical symptom in SLE patients; hence, if a patient has such a problem, SLE should be suspected, and related diagnostic tests should be considered to improve patient outcomes and prevent disease progression. Due to the variety of clinical symptoms of SLE in various geographical and ethnic groups, defining the typical pattern of disease for a correct diagnosis is strongly recommended.

## CRediT authorship contribution statement

**Leila Soltani:** Data curation, Methodology, Writing – original draft. **Ava Hashempour:** Conceptualization, Funding acquisition, Supervision, Writing – original draft, Writing – review & editing. **Javad Moayedi:** Conceptualization, Formal analysis, Methodology, Software, Writing – review & editing. **Maryam Feili:** Data curation, Methodology, Writing – review & editing. **Zahra Musavi:** Data curation, Methodology, Writing – review & editing. **Mohammad Ali Nazarinia:** Conceptualization, Data curation, Methodology, Writing – review & editing.

## Declaration of competing interest

The authors declare that they have no known competing financial interests or personal relationships that could have appeared to influence the work reported in this paper.

## References

[1] Rezvaninejad R., Dadmehr M., Rezvaninejad R. (2021). Prevalence of oral manifestations in systemic lupus erythematosus patients Referred to shahid mohammadi hospital in 2018-2019. Jundishapur J Health Sci.

[2] Klein A., Polliack A., Gafter-Gvili A. (2018). Systemic lupus erythematosus and lymphoma: incidence, pathogenesis and biology. Leuk Res.

[3] Sun Y., Sun S., Li W., Li B., Li J. (2011). Prevalence of human herpesvirus 8 infection in systemic lupus erythematosus. Virol J.

[4] Qin L., Qiu Z., Hsieh E., Geng T., Zhao J., Zeng X. (2019). Association between lymphocyte subsets and cytomegalovirus infection status among patients with systemic lupus erythematosus: a pilot study. Medicine.

[5] Münz C., Lünemann J.D., Getts M.T., Miller S.D. (2009). Antiviral immune responses: triggers of or triggered by autoimmunity?. Nat Rev Immunol.

[6] Hashempour A., Moayedi J., Musavi Z., Ghasabi F., Halaji M., Hasanshahi Z. (2021). First report of HHV-8 viral load and seroprevalence of major blood-borne viruses in Iranian patients with systemic sclerosis. Mult Scler Relat Disord.

[7] Sadeghifar J., Jalilian H., Momeni K., Delam H., Sheleme T., Rashidi A. (2021). Outcome evaluation of COVID-19 infected patients by disease symptoms: a cross-sectional study in Ilam Province, Iran. BMC Infect Dis.

[8] Ocampo-Piraquive V., Nieto-Aristizábal I., Cañas C.A., Tobón G.J. (2018). Mortality in systemic lupus erythematosus: causes, predictors and interventions. Expet Rev Clin Immunol.

[9] Aringer M., Hiepe F. (2011). Systemic lupus erythematosus. Z Rheumatol.

[10] Schwartzman-Morris J., Putterman C. (2012). Gender differences in the pathogenesis and outcome of lupus and of lupus nephritis. J Immunol Res.

[11] Kone-Paut I., Piram M., Guillaume S., Tran T.A. (2007). Lupus in adolescence. Lupus..

[12] Aggarwal A., Srivastava P. (2015). Childhood onset systemic lupus erythematosus: how is it different from adult SLE?. Int J Rheum Dis.

[13] Massias J.S., Smith E.M., Al-Abadi E., Armon K., Bailey K., Ciurtin C. (2021). Clinical and laboratory phenotypes in juvenile-onset Systemic Lupus Erythematosus across ethnicities in the UK. Lupus.

[14] Sitnik R., Pinho J.R.R., Bertolini D.A., Bernardini A.P., Da Silva L.C., Carrilho F.J. (2004). Hepatitis B virus genotypes and precore and core mutants in Brazilian patients. J Clin Microbiol.

[15] Pour M.A., Keivani H., Sabahi F., Alavian S. (2006). Determination of HCV genotypes in Iranian isolates by PCR-RFLP. Iran J Public Health.

[16] Gentile I., Zappulo E., Coppola N., Bonavolta R., Portella G., Cernia D.S. (2013). Prevalence of HHV-6 and HHV-8 antibodies in patients with autism spectrum disorders. In Vivo.

[17] Geraminejad P., Memar O., Aronson I., Rady P.L., Hengge U., Tyring S.K. (2002). Kaposi's sarcoma and other manifestations of human herpesvirus 8. J Am Acad Dermatol.

[18] Rider J., Ollier W., Lock R., Brookes S., Pamphilon D. (1997). Human cytomegalovirus infection and systemic lupus erythematosus. Clin Exp Rheumatol.

[19] James J.A., Neas B.R., Moser K.L., Hall T., Bruner G.R., Sestak A.L. (2001). Systemic lupus erythematosus in adults is associated with previous Epstein‐Barr virus exposure. Arthritis Rheum.

[20] Draborg A.H., Duus K., Houen G. (2013). Epstein-Barr virus in systemic autoimmune diseases. Clin Dev Immunol.

[21] Wang S., Chen Y., Xu X., Hu W., Shen H., Chen J. (2017). Prevalence of hepatitis B virus and hepatitis C virus infection in patients with systemic lupus erythematosus: a systematic review and meta-analysis. Oncotarget.

[22] Kaye B.R. (1996). Rheumatologic manifestations of HIV infections. Clin Rev Allergy Immunol.

[23] Nazarinia M., Ghaffarpasand F., Shamsdin A., Karimi A., Abbasi N., Amiri A. (2008). Systemic lupus erythematosus in the fars province of Iran. Lupus.

[24] Mahmoudi Z., Nikjoo M., Rezaiemanesh A., Ahmadi M., Pourmand D. (2021). Evaluation of demographic, clinical and laboratory features of patients with systemic lupus erythematosus in kermanshah. J Clin Res Paramed Sci..

[25] Akbarian M., Faezi S.T., Gharibdoost F., Shahram F., Nadji A., Jamshidi A.R. (2010). Systemic lupus erythematosus in Iran: a study of 2280 patients over 33 years. Int J Rheum Dis.

[26] Barcia‐Sixto L., Isenberg D. (2020). Systemic lupus erythematosus: causes and manifestations. Trends Urol Men's Health.

[27] Dubois E.L., Tuffanelli D.L. (1964). Clinical manifestations of systemic lupus erythematosus: computer analysis of 520 cases. J Am Med Assoc.

[28] Mahmoudi M., Rastin M., Sahebari M., Zamani S., Tabasi N. (2017). Autoantibody profile, disease activity and organ involvement in Iranian systemic lupus erythematosus patients. Rheumatol Res.

[29] Bonakdar Z.S., Nasiri S., Karimifar M., Karimzadeh H., Salesi M., Motaghi P. (2012). Clinical and laboratory signs among systemic lupus erythematosus patients in isfahan. J Isfahan Med Sch.

[30] Yaghoobi R., Fathi J. (2000). Cutaneous manifestations of systemic lupus erythematosus: a study from ahwaz. Iranian J Dermatol.

[31] Ashournia P., Sadeghi P., Rezaei N., Moradinejad M.-H., Ziaee V. (2018). Prevalence of family history of autoimmune disorders in juvenile systemic lupus erythematosus. Maedica.

[32] Mohammadoo-Khorasani M., Salimi S., Tabatabai E., Sandoughi M., Zakeri Z., Farajian-Mashhadi F. (2016). Interleukin-1β (IL-1β) & IL-4 gene polymorphisms in patients with systemic lupus erythematosus (SLE) & their association with susceptibility to SLE. Indian J Med Res.

[33] Cervera R., Khamashta M.A., Font J., Sebastiani G.D., Gil A., Lavilla P. (1993). Systemic lupus erythematosus: clinical and immunologic patterns of disease expression in a cohort of 1,000 patients. The European Working Party on Systemic Lupus Erythematosus. Medicine.

[34] Mok C., Lau C. (2003). Lupus in Hong Kong Chinese. Lupus.

[35] Habib G.S., Saliba W.R. (2002). Systemic lupus erythematosus among Arabs. Isr Med Assoc J.

[36] Rastin M., Hatef M.R., Tabasi N., Mahmoudi M. (2012). The pathway of estradiol-induced apoptosis in patients with systemic lupus erythematosus. Clin Rheumatol.

[37] Dhital R., Guma M., Poudel D.R., Chambers C., Kalunian K. (2023). Infection-related hospitalisation in young adults with systemic lupus erythematosus: data from the National Inpatient Sample. Lupus Sci Med.

[38] Restrepo-Escobar M., Castaño-González P., Galvis-García M., Morales-Maya L., Urrego T., Sandoval-Álvarez S. (2021). Development and internal validation of a clinical prediction model of the risk of nosocomial bacterial infection in patients with systemic lupus erythematosus. Rev Colomb Reumatol.

[39] Beltran A., Moreno Lopez S., Vega-Hoyos D., Lesmes A., Calderón O., Uribe S. (2021). Infection detection in patients with systemic lupus erythematosus using a hospital administrative database. Rev Colomb Reumatol.

[40] Segura B.T., Rua-Figueroa I., Pego-Reigosa J.M., Del Campo V., Wincup C., Isenberg D. (2019). Can we validate a clinical score to predict the risk of severe infection in patients with systemic lupus erythematosus? A longitudinal retrospective study in a British Cohort. BMJ Open.

[41] Mehta P., Singh K., Anand S., Parikh A., Patnaik A., Chatterjee R. (2022). Differentiating flare and infection in febrile lupus patients: derivation and validation of a calculator for resource constrained settings. Lupus.

[42] Illescas‐Montes R., Corona‐Castro C.C., Melguizo‐Rodríguez L., Ruiz C., Costela‐Ruiz V.J. (2019). Infectious processes and systemic lupus erythematosus. Immunology.

